# *N*-acetylcysteine potentiates doxorubicin-induced ATM and p53 activation in ovarian cancer cells

**DOI:** 10.3892/ijo.2012.1680

**Published:** 2012-10-26

**Authors:** GABRIELLA BRUM, THOMAS CARBONE, ERIC STILL, VENDITA CORREIA, KEVIN SZULAK, DAVID CALIANESE, CHARLES BEST, GARRET CAMMARATA, KATELYN HIGGINS, FANG JI, WEN DI, YINSHENG WAN

**Affiliations:** 1 Department of Biology, Providence College, Providence, RI 02918, USA;; 2 Department of Obstetrics and Gynecology, Renji Hospital of Shanghai Jiaotong University Medical School, Shanghai 200001, P.R. China

**Keywords:** *N*-acetylcysteine, ATM, p53, doxorubicin, ovarian cancer

## Abstract

Doxorubicin has been used clinically to treat various types of cancer, and yet the molecular mode of actions of doxorubicin remains to be fully unraveled. In this study, we investigated the effect of doxorubicin on cultured ovarian cancer cells (CaOV3). MTT assay data showed that doxorubicin inhibits cell proliferation in a time- and dose-dependent manner. Phagokinetic cell motility assay data indicated that doxorubicin inhibits both basal level and EGF-induced cell migration in CaOV3 cells. Confocal microscopic data revealed that doxorubicin induces reorganization of cytoskeletal proteins including actin, tubulin and vimentin. Doxorubicin induces phosphorylation of p53 at Ser15 and 20, acetylation of p53 and ATM activation. Doxorubicin also induces phosphorylation of histone H2AX at Ser139. Interestingly, doxorubicin also inhibits mTOR activity, measured by phosphorylation of S6 ribosomal protein. Pretreatment of CaOV3 cells with antioxidant *N*-acetylcysteine (NAC), but not pyrrolidine dithiocarbamate (PDTC) potentiates doxorubicin-induced phosphorylation of p53 and ATM. Collectively, we conclude that doxorubicin induces ATM/p53 activation leading to reorganization of cytoskeletal networks, inhibition of mTOR activity, and inhibition of cell proliferation and migration. Our data also suggest that removal of oxidants by antioxidants such as NAC may enhance the efficacy of doxorubicin *in vivo*.

## Introduction

Doxorubicin (Dox) is a potent antineoplastic agent used to treat various types of cancer including ovarian cancer, despite a cumulative cardiomyopathy that might reduce the therapeutic index for treatment (1). It has been shown that doxorubicin causes DNA damage, with a result of cell cycle block or apoptosis (2), while other studies support the notion that different doses of doxorubicin activate different regulatory mechanisms to induce either apoptosis or cell death through mitotic catastrophe (3).

ATM/p53 pathway is involved in Dox-induced apoptosis. Studies have demonstrated that pretreatment of cells with the hydroxyl radical scavenger, *N*-acetylcysteine (NAC) significantly attenuates doxorubicin-mediated phosphorylation and accumulation of p53, p53-DNA binding, and the phosphorylation of H2AX and Cdk4, suggesting that hydroxyl radicals contribute to the doxorubicin-induced activation of ATM-dependent pathways (4,5).

Studies have also shown that wortmannin, an inhibitor of ATM that has high homology with PI3K family and is located upstream of p53, inhibits activation of Cdk4 without the induction of p53 in Dox-induced cell death (6). These data suggest that Cdk4 is one of the essential components for induction of cell death; p53 may prevent Dox-induced cell death through p21; and Cdk4 may be activated by ATM, which is necessary for the activation of p53 in Dox-induced apoptosis (6).

In addition to ATM/p53 pathway activation, accumulating evidence suggest that Dox activates distinct members of PI3K family which then activates MAPK/NF-κB cell survival pathway that opposes the apoptotic response following DNA damage (7) and may eventually contribute to drug resistance. Abundant studies have demonstrated that activation of PI3K/AKT pathway leading to mTOR activation with a result of activation of downstream component such as s6 ribosomal protein (8). However, the question as to whether Dox has any effect on mTOR remains to be addressed.

The cytoskeleton plays an important role in many cellular processes, including mitosis, cytokinesis, intracellular transport, endocytosis and secretion, and it is also involved in the transcriptional activities of various genes. Induction of cell death with doxorubicin treatment is has been shown to be associated with both mitotic catastrophe (at both lower and higher doxorubicin concentrations) and apoptosis (at higher doxorubicin concentration). It has been proposed that apoptosis and mitotic catastrophe are independent from each other (9).

Studies have shown that doxorubicin induces re-distribution of F-actin, vimentin and tubulin (10). The reorganization of cytoskeletal proteins is consistent with features of apoptosis, with an increase in bright staining of F-actin, vimentin and tubulin at the site of apoptotic body formation, suggesting that actin might be involved in the chromatin remodeling during apoptosis (10). However, whether Dox induces cytoskeletal protein reorganization and chromatin remodeling in ovarian cancer cells remains unknown.

Cell migration is regulated by actin and microtubule cytoskeleton in which Rho GTPases are involved. It has been suggested that targeting actin cytoskeleton by cancer drugs may provide a means to prevent metastasis formation (1,11). Our preliminary studies have shown that Dox inhibits ovarian cancer cell migration. However, the question whether Dox-inhibited cell migration is related to cytoskeletal protein reorganization remains to be addressed.

Given that cytoskeletal proteins play important roles in apoptosis and cell migration, we undertook this project to study the effect of doxorubicin on both apoptosis and cell migration via cytoskeletal protein reorganization and ATM/p53 pathway activation. Our data showed that doxorubicin-induced cell death and inhibition of cell migration are associated with cytoskeletal protein reorganization, ATM/p53 activation, and chromatin remodeling. Antioxidant NAC potentiates the effect of doxorubicin *in vitro* in cultured ovarian cancer cells (CaOV3 cells) and may be applied *in vivo* studies.

## Materials and methods

### Chemicals and reagents

Doxorubicin, NAC, pyrrolidine dithiocarbamate (PDTC) and monoclonal mouse anti-β-actin were obtained from Sigma (St. Louis, MO, USA). Wortmannin and Hoechst were from CalbioChem (San Diego, CA, USA). rabbit anti-tubulin, rabbit anti-vimentin, rabbit anti-p-p53, acetylated p53, p-ATM and p-H2AX were from Cell Signaling Technology (Beverly, MA, USA). Alexa Fluor^®^ 488 goat anti-rabbit/mouse IgG (H+L), Alexa Fluor^®^ 680 goat anti-rabbit/mouse IgG (H+L), were from Life Technologies (Grand Island, NY, USA).

### Cell culture

Human ovarian cancer CaOV3 cells were maintained in DMEM (Sigma) supplemented with 10% fetal bovine serum (FBS), penicillin/streptomycin (1:100, Sigma) and 4 mM L-glutamine, in a CO_2_ incubator at 37°C.

### Cell viability assay (MTT dye assay)

Cell viability was measured by the 3-[4,5-dimethylthylthiazol-2-yl]-2,5 diphenyltetrazolium bromide (MTT) method as previously described (12). Briefly, cells were collected and seeded in 96-well plates at a density of 2×10^5^ cells/cm^2^. After incubation for 24 h, cells were exposed to fresh medium containing reagents at 37°C. After incubation for certain period, 20 *μ*l of MTT tetrazolium (Sigma) salt dissolved in PBS at a concentration of 5 mg/ml was added to each well and incubated in CO_2_ incubator for 4 h. Finally, the medium was aspirated from each well and 150 *μ*l of DMSO (Sigma) was added to dissolve formazan crystals and the absorbance of each well was obtained using a Dynatech MR5000 plate reader at a test wavelength of 490 nm with a reference wavelength of 630 nm.

### Cell migration assay

The phagokinetic track motility assay was performed to determine cell migration as previously described (13–15). Briefly, 12-well plates were coated with 20 *μ*g/ml of fibronectin (Sigma) and 2.4 ml of microsphere suspension (86 *μ*l of stock microbead solution in 30 ml of PBS, Sigma) was added to each well. The plates were then centrifuged at 1,200 rpm at 4°C for 20 min and carefully transferred to a CO_2_ incubator at 37°C for at least 1 h. About 1.8 ml of supernatant was removed from each well and finally 1,500 freshly trypsinized cells in 2 ml of DMEM were seeded in each well. Cells were cultured for 36 h in the presence or absence of the appropriate reagents and then photographed with an inverted microscope with a Carl Zeiss digital camera (SPOT). At least 50 cells in 10 random views from each condition were quantified for the distance that they had migrated.

### Confocal microscopy

Cells were plated in 8-well chamber slides (Lab-Tek, Nalge Nunc International, Naperville, IL, USA) and washed in phosphate balanced saline (PBS) to remove traces of medium. The cells were then fixed for 20 min in fresh 4% paraformaldehyde-PBS, permeabilized and blocked with normal goat serum (diluted 1:10) for 2 h in PBS. Cells were then washed three times in PBS and incubated with primary antibody overnight at room temperature, followed by another three times PBS wash and secondary antibody incubation at room temperature for 1 h. The cell nuclei were also stained with Hoechst (1 *μ*g/ml in PBS) for 10 min. The slides were mounted with anti-fade (Life Technologies, Grand Island, NY, USA) and kept in the dark until viewing. The samples were observed under a confocal microscope (Carl Zeiss) and images were captured by Zen 2009 Light Edition.

## Results

### Doxorubicin induces cell death in cultured ovarian cancer cells

**Figure 1.**
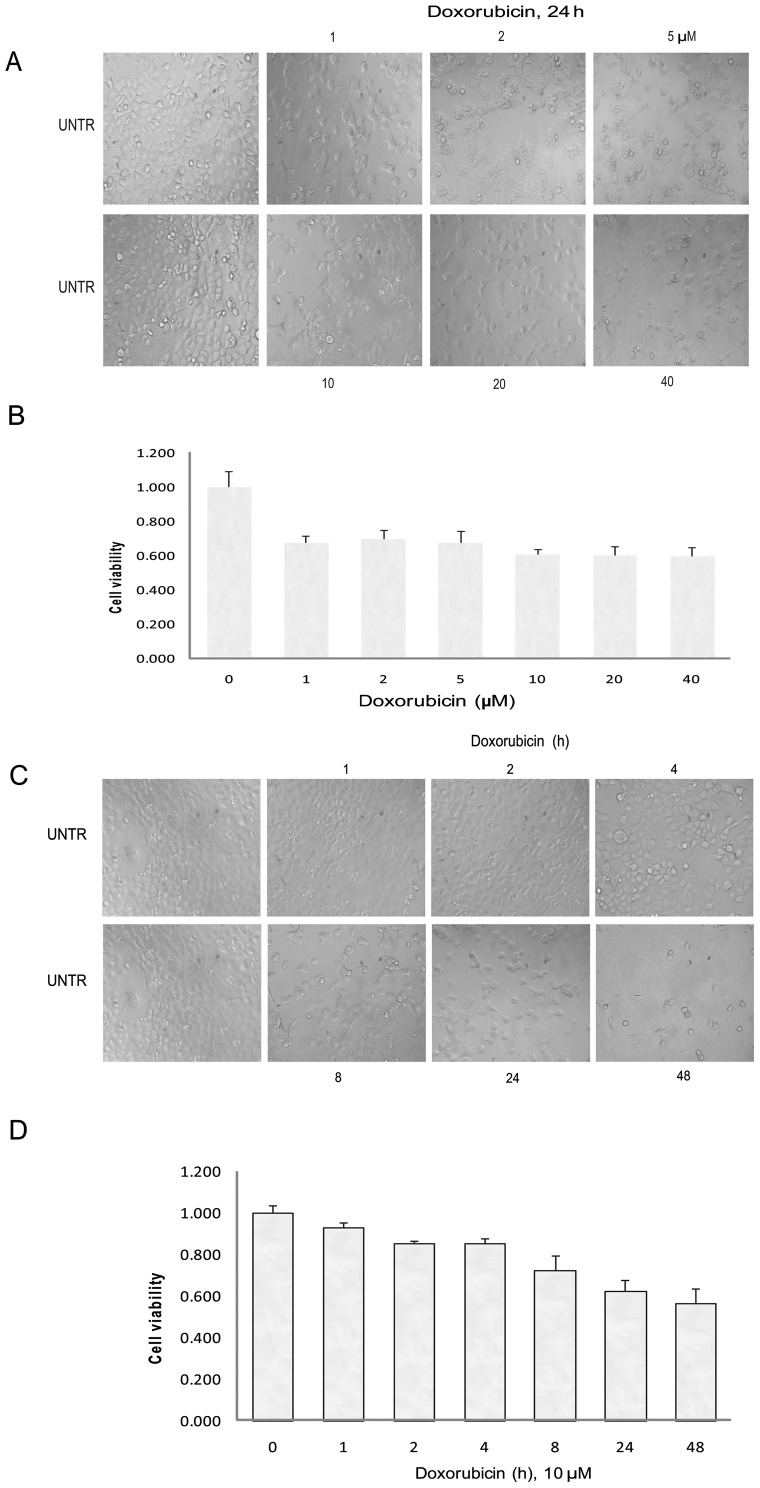
Effect of doxorubicin on cell viability in cultured ovarian cancer cells. CaOV3 cells were cultured in 96-well plates and treated with various concentrations of doxorubicin. (A) Cells were observed 24 h after treatment under an inverted microscope and (B) cell viability was measured by MTT assay. Cells were treated with doxorubicin at the concentration of 10 *μ*M and (C) observed at different time points and (D) cell viability was assayed by MTT.

Doxorubicin has been shown to induce cell death in a variety of cancer cells including ovarian cancer cells *in vitro*(16,17) and clinically applied to various types of cancer (18). Different doses of doxorubicin activate different regulatory mechanisms to induce either apoptosis or cell death through mitotic catastrophe in cancer cells (3). To test the effect of doxorubicin on cell death in cultured ovarian cancer cells, we cultured CaOV3 cells in 96-well plates and treated the cells with various concentrations of doxorubicin or at different time points. The results showed that doxorubicin induces cell death in a time- and dose-dependent manner (Fig. 1A–D).

### Doxorubicin inhibits EGF-induced cell migration in cultured ovarian cancer cells

**Figure 2.**
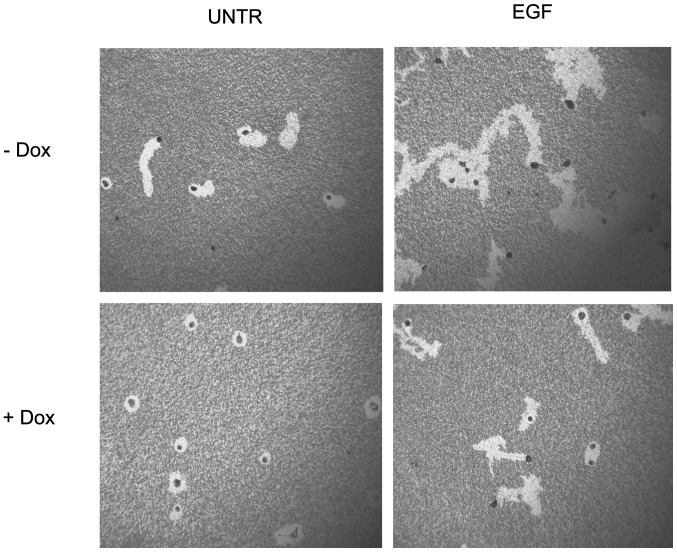
Effect of doxorubicin on ovarian cancer cell migration. CaOV3 cells were cultured and treated with or without doxorubicin or EGF and cell migration was monitored 24 h after treatment by phagokinetic mobility track assay.

Above data show that doxorubicin inhibits cell proliferation in cultured ovarian cancer cells. Our previous studies have shown that EGF induces cell migration in CaOV3 cells, which is inhibited by EGFR inhibitors and curcumin (19). To test whether Doxorubicin has any effect on cell migration, we used phagokinetic track motility assay. The results showed that doxorubicin inhibits cell migration with or without EGF treatment (Fig. 2).

### Doxorubicin induces reorganization of cytoskeletal proteins in CaOV3 cells

**Figure 3.**
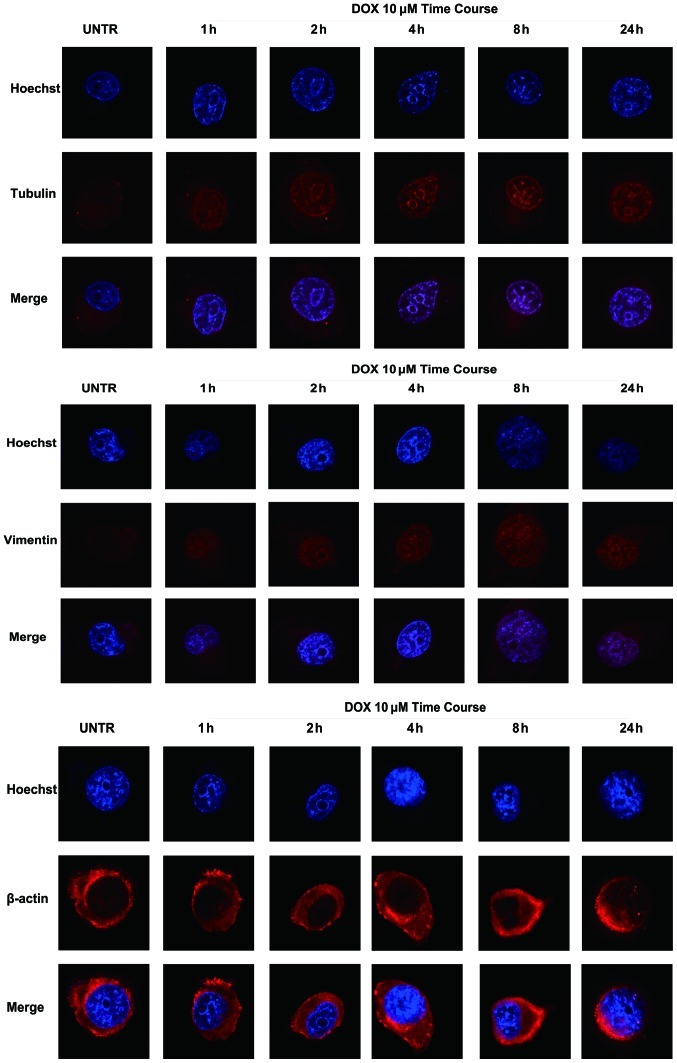
Effect of doxorubicin on cytoskeletal proteins in cultured ovarian cancer cells. CaOV3 cells were cultured in 8-well chamber slides, treated with 10 *μ*M of doxurubicin and fixed at different time points. Tubulin, vimentin and actin filaments were stained with antibodies and visualized under a confocal microscope.

The cytoskeleton plays an important role in many cellular processes but also is involved in gene transcription. Interference with actin dynamics is superior to disturbance of microtubule function in the inhibition of human ovarian cancer cell motility (11). Doxorubicin induces morphological alterations in mitochondrial, nuclear and fibrous protein structures, which are dependent on the drug concentration (20–22). Doxorubicin affects actin assembly *in vitro*(9,23,24). To study the effect of doxorubicin on cytoskeletal proteins, we cultured CaOV3 cells in 8-well chamber slides. The cells were treated with 10 *μ*M of doxorubicin and fixed at various time points, and stained with anti-actin, tubulin and vimentin antibodies. Confocal microscopic data showed that doxorubicin treatment induces reorganization of cytoskeletal proteins (Fig. 3).

### NAC potentiates doxorubicin-induced activation of ATM, p53, and histone proteins

**Figure 4.**
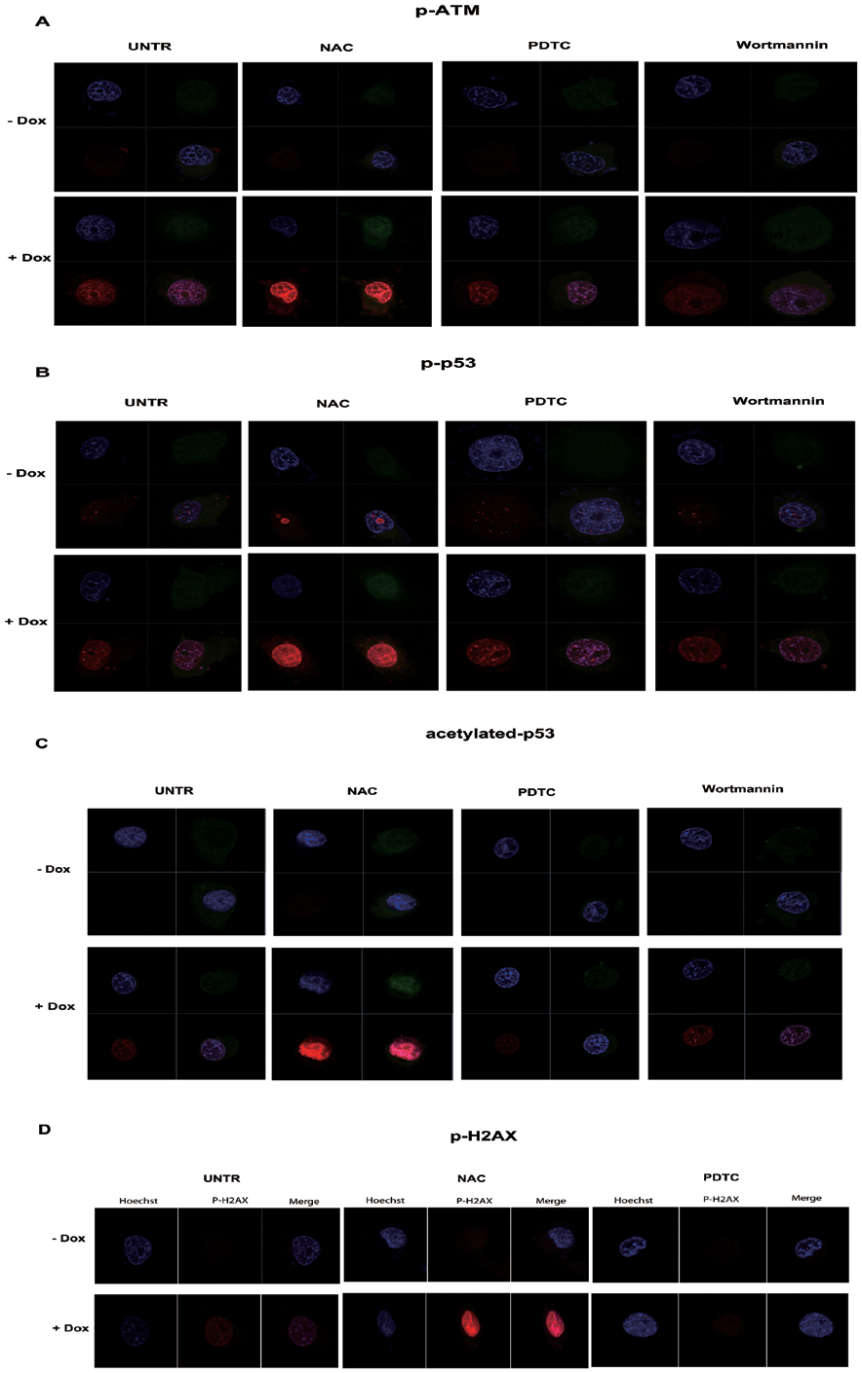
Effect of NAC on doxorubicin-induced activation of ATM, p53 and H2AX in cultured ovarian cancer cells. CaOV3 cells were cultured in 8-well chamber slides, pretreated with NAC, PDTC, or Wortmannin, for 2 h and then treated with doxorubicin. Cells were fixed 2 h post-doxorubicin treatment. Antibodies were used to detect (A) phospho-ATM, (B) phospho-p53, (C) acetylated p53 and (D) phospho-H2AX. Confocal microscopy was performed.

ATM/p53 pathway has been reported to be involved in apoptosis of various cancer cells induced by chemotherapy drugs (25). Doxorubicin has been shown to activate ATM and p53, leading to cell death, mediated by reactive oxygen species (4,26,27). Enhanced microtubule-dependent trafficking and p53 nuclear accumulation by suppression of microtubule dynamics (28). Chromosomal breaks during mitotic catastrophe trigger γ-H2AX-ATM-p53-mediated apoptosis (29). Antioxidant NAC has been shown to affect doxorubicin-induced cell death in two distinct ways (30). To test whether NAC has effect on Dox-induced ATM and p53 activation, we treated the cells with NAC (500 *μ*M) for 2 h, then Dox for 2 h. Cells were stained with anti-p-ATM and p-p53. Confocal microscopic data showed that Dox induces ATM and p53 phosphorylation. However, surprisingly, NAC pretreatment enhances ATM and p53 phosphorylation induced by Dox. Neither PTDC nor Wortmannin has such effect (Fig. 4A and B). The results also showed that doxorubicin induces p53 acetylation and H2AX phosphorylation, which is also enhanced by NAC but not PDTC or Wortmannin (Fig. 4C and D).

## Discussion

Doxorubicin has been used clinically to treat various types of cancer, and the molecular mechanisms through which doxorubicin acts are still being elucidated. Studies have suggested that induction of cell death with doxorubicin treatment is due to two independent events, namely mitotic catastrophe and apoptosis, depending on the treated dose (9). Our present study shows that in addition to apoptosis induction, doxorubicin also inhibits cell migration in ovarian cancer cell line CaOV3 cells (Figs. 1 and 2).

Studies have been done to investigate the effect of doxorubicin on reorganization of F-actin, vimentin and tubulin during apoptosis (10). The reorganization of cytoskeletal proteins appears to be linked to features of apoptosis. The results that F-actin, vimentin and tubulin are remarkably concentrated at the apoptotic bodies suggest that these cytoskeletal proteins play important roles in this process. In this study, we found that doxorubicin induces cytoskeletal protein reorganization in ovarian cancer CaOV3 cells. This reorganization may contribute to the apoptosis of cells induced by doxorubicin.

It has been shown that cell movement and migration are mainly orchestrated by the actin and microtubule cytoskeleton. Various cytoskeleton modifications are associated with malignant cell transformation and have been used as prognostic factors. Targeting cytoskeleton network may attenuate metastatic activities of tumor cells (11,31). In addition to actin cytoskeletal proteins, research has suggested that intermediate filament vimentin could be a marker associated with chemoresistance or a marker of malignancy in certain epithelial cancers (32). Changes in vimentin filaments has been reported in dox treated leukemia cells (10,33–35). Vimentin mediate a drug-resistant invasive phenotype in diffuse large B cell lymphoma (36). We found that vimentin is also reorganized in doxorubicin treated CaOV3 cells (Fig. 3).

ATM/p53 pathway has been reported to be involved in apoptosis of various cancer cells induced by chemotherapy drugs (25). Doxorubicin has been shown to activate ATM and p53, leading to cell death (4,26,27). Doxorubicin enhances microtubule-dependent trafficking and p53 nuclear accumulation by suppression of microtubule dynamics (28). Chromosomal breaks during mitotic catastrophe induces H2AX-ATM-p53-mediated apoptosis (29). In this study, we observed that doxorubicin induces ATM and p53 phosphorylation (Fig. 4A and B) in CaOV3 cells. Also, our results showed that doxorubicin induces p53 acetylation and H2AX phosphorylation (Fig. 4C and D).

Antioxidant *N*-acetylcysteine, has been shown to affect doxorubicin-induced cell death in two distinct ways (30). NAC, a pro-glutathione drug, increases the resistance of both cells against doxorubicin. Studies have demonstrated that NAC enhances MRP1-mediated doxorubicin resistance and this effect depends on GSH synthesis (37). It has been shown that doxorubicin induces ATM activation via production of reactive oxygen species and antioxidants, such as NAC, inhibiting doxorubicin-induced ATM activation (4). In this study, we tested whether NAC has any effects on doxorubicin-induced ATM activation in CaOV3 cells. Surprisingly, NAC pretreatment enhances ATM and p53 phosphorylation, p53 acetylation and H2AX phosphorylation (Fig. 4). Interestingly, neither PDTC nor wortmannin has this effect.

Cancer cells including ovarian cancer cells are resistant to chemotherapy drugs such as doxorubicin. It would be beneficial to chemotherapy if such resistance is suppressed. NAC has been shown to protect from doxorubicin-induced hepatotoxicity and cardiotoxicity during chemotherapy (30,38–40). NAC also has been shown to enhance drug resistance (37). However, according to our data, NAC may potentiate doxurubicin’s effect in chemotherapy in ovarian cancer. In fact, combinatory therapy with doxorubicin has been becoming more beneficial approach (41,42). Further *in vitro* and *in vivo* studies will be necessary to test such possibility.
